# Independent Validation and Refinement of the *LAX1*-Regulated Transcriptional Network in Rice Panicle Development

**DOI:** 10.3390/plants15192923

**Published:** 2026-09-24

**Authors:** Jin-Lin Bao, Han Li, Jian-Xin Wei, Yu-Xian Huang, Jia-Tong He, Jian Jin, Jing Huang

**Affiliations:** 1 State Key Laboratory for Conservation and Utilization of Subtropical Agro-Bioresources, Guangxi University, Nanning 530004, China; 2 College of Agriculture, Guangxi University, Nanning 530004, China; 3 College of Life Science and Technology, Guangxi University, Nanning 530004, China

**Keywords:** rice, *LAX1*, panicle architecture, transcriptomics, auxin transport, *PIN* genes, CRISPR/Cas9

## Abstract

Rice panicle architecture plays a critical role in determining grain yield; however, the regulatory networks influencing axillary meristem formation remain inadequately elucidated. A previous study identified *LAX1* as a canonical bHLH transcription factor, detailing its direct targets and global transcriptomic alterations in *lax1* mutants. In this study, an independent CRISPR/Cas9 *LAX1* knockout mutant was generated, followed by transcriptome profiling of young panicles. The *lax1*-KO mutant displayed a reduction in secondary branches and grain number. Applying a more stringent threshold (|log_2_ fold change (log_2_FC)| ≥ 1), 518 high-confidence differentially expressed genes (DEGs) were identified, 131 of which overlapped with previously reported DEGs (direction concordance: 88.5%), supporting the core *LAX1*-regulated network. The analysis revealed member-specific *PIN* regulation (*OsPIN1c/1d* and *OsPIN2* downregulated; *OsPIN3t* and *OsPIN9* upregulated), expression changes in hormone-associated genes (*OsPP2C09*, *OsPP2C49*, *OsRR3*, *OsRR9*, *OsGASR9*) and *OsTPP* genes (*OsTPP1*, *OsTPP4*, *OsTPP9*), and altered expression of panicle regulators (*IPA1*, *OsTB1*, *DEP1*, *DEP3*, *CUC*, and MADS-box genes). Motif enrichment analysis further identified bHLH motifs as the most frequently represented, whereas CAMTA and FRS/FRF motifs exhibited the highest statistical significance in DEG promoters, suggesting a possible multi-layered transcriptional architecture. Collectively, these findings validate and refine the *LAX1*-regulated network, positioning *LAX1* as a pivotal coordinator of rice panicle development.

## 1. Introduction

Rice (*Oryza sativa* L.) serves as a staple food for over half of the global population and represents a fundamental model for cereal biology [1]. Grain yield is significantly influenced by panicle architecture, characterized by rachis length, branch number, and spikelet arrangement [2]. Panicle development occurs through the maintenance of the shoot apical meristem (SAM), its transition to an inflorescence meristem (IM), and the subsequent initiation of the axillary meristem (AM), which gives rise to branches and spikelets [3,4]. These processes are intricately regulated by a complex network of transcription factors and phytohormones, particularly auxin and cytokinin, which play important roles in meristem activity and branching [5,6,7,8,9,10].

Genetic studies have identified multiple regulators involved in rice panicle development, classifying these genes into three categories based on their phenotypic effects: panicle branch and lateral spikelets, multifloret spikelets, and panicle type [6]. The first category includes genes controlling meristem initiation and branch formation, such as *LAX PANICLE 1* (*LAX1*), *LAX PANICLE 2* (*LAX2*), *MONOCULM 1* (*MOC1*), and *FRIZZY PANICLE* (*FZP*) [9,11,12,13]. It also encompasses genes regulating branch outgrowth and panicle architecture, including *IDEAL PLANT ARCHITECTURE 1* (*IPA1*), *DENSE AND ERECT PANICLE 1* (*DEP1*), *DEP3*, and *OsTB1*/*FC1* [14,15,16,17]. The *CUP-SHAPED COTYLEDON* genes (*OsCUC1* and *OsCUC3*) contribute to this process by defining meristem and organ boundaries [18]. The second category, multifloret spikelets, consists of genes such as G1 and MADS-box genes, which dictate spikelet meristem identity and floral organ development [3,19]. The third category, panicle type, includes genes that govern the transition from branch meristems to spikelet meristems and subsequent panicle establishment, exemplified by *ABERRANT PANICLE ORGANIZATION 1 (APO1*), *APO2/RFL*, *TAWAWA1* (*TAW1*) and *PANICLE DEVELOPMENT 8* (*PCD8*) [20,21,22]. Additionally, *FZP2*, along with *SP1* and *GNP1*, influences panicle length and grain number [13,23,24,25]. Hormone-related genes—including *CYTOKININ OXIDASE 2* (*OsCKX2*)/*Gn1a*, *LONELY GUY* (*LOG*), *SUPPRESSOR of EFFECTIVE PANICL 1* (*SEP1*) and *PINOID* (*OsPID*)—modulate cytokinin and auxin homeostasis, thereby critically influencing meristem activity and panicle architecture [5,26,27,28]. Despite these advancements, the hierarchical organization and molecular interactions within this regulatory network remain inadequately understood.

Population-level transcriptome-wide association analyses have recently identified *LAX1* as a hub gene whose targets are significantly enriched for panicle-related quantitative trait loci, highlighting its important role in the panicle regulatory network [29]. *LAX1* encodes a basic helix–loop–helix (bHLH) transcription factor, functioning as a key regulator of AM initiation during rice inflorescence development [9,11]. Loss-of-function lax1 mutants exhibit significant defects in panicle branching, characterized by reduced primary and secondary branches, alongside notably lower grain yield [13]. *LAX1* is expressed at the boundary between IM and nascent AMs, with its protein moving non-cell-autonomously to adjacent AM initiation zones [11]. Genetically, *LAX1* collaborates with multiple known regulators: it synergizes with *LAX2* and *MOC1*, as evidenced by severely exacerbated branching defects in *lax1lax2* and *lax1moc1* double mutants [12]. In contrast, *LAX1* operates independently of *FZP*, as the *lax1fzp* double mutant presents additive phenotypes [13]. Additionally, *LAX1* is positively regulated by *RFL* and negatively regulated by miR156 at the transcriptional level [30,31]. At the transcriptional level, *LAX1* expression is also directly repressed by *OsSPL7* through binding to GTAC motifs in its promoter [32]. At the protein level, LAX1 physically interacts with LAX2 [12], OsbHLH067/068/069—redundantly regulating AM formation [33]—and MADS-box proteins OsMADS1/6/7 during floral organ development [34]. Natural variation at the *LAX1* locus has been shown to affect yield-related traits, highlighting its agronomic importance [35,36].

*LAX1* and its orthologs, including BARREN STALK FASTIGIATE1 (BA1) in maize, REGULATOR OF AXILLARY MERISTEM FORMATION (ROX) in Arabidopsis, and TaLAX1 in wheat, belong to the VIIIb subfamily of bHLH transcription factors [37]. Initially classified as a “low-basic” bHLH protein due to containing fewer than five basic amino acids in its basic region, LAX1 was presumed to lack intrinsic DNA-binding capability [38]. This assumption has recently been reconsidered, as a study showed that LAX1 possesses intrinsic DNA-binding ability and directly binds to the promoters of target genes, including OsPID, OsIAA7, LOG, and OsTPS8 [39]. Transcriptome analysis further revealed that LAX1 broadly impacts hormone and metabolic pathways, thereby establishing it as a canonical bHLH transcription factor. Nevertheless, a comprehensive understanding of how the loss of LAX1 reshapes specific branches of hormone signaling, particularly concerning the auxin transporter network and downstream meristem identity regulators, remains incomplete. To further investigate this, an independent CRISPR/Cas9 LAX1 knockout mutant was generated, followed by transcriptome profiling of young panicles. Applying a more stringent threshold aimed at identifying a high-confidence set of differentially expressed genes (DEGs) offers a systems-level perspective that independently supports and extends the transcriptomic framework established by a recent study.

## 2. Results

### 2.1. Generation of LAX1-KO Lines by CRISPR/Cas9

*LAX1* (LOC_Os01g61480) knockout lines were generated in the rice cultivar Zhonghua 11 (ZH11) using CRISPR/Cas9, targeting its sole exon (Figure 1A). A total of 19 T_0_ transgenic plants were obtained, and the target site was analyzed in all samples. Sanger sequencing of the target amplicon, complemented by DsDecodeM analysis [40], identified 6 non-knockout (non-KO) plants with an intact *LAX1* allele and 13 knockout (KO) plants exhibiting either a 1-bp insertion (T or C) or a deletion (Table S1). A homozygous *lax1*-KO line with a single C deletion at position 51 bp was selected, resulting in a frameshift mutation and premature termination of the LAX1 protein (Figure 1A). Off-target analysis using CRISPR-P v2.0. Sanger sequencing of the top five predicted off-target loci confirmed that no mutations were introduced at these sites (Table S2). Quantitative reverse-transcription PCR (qRT-PCR) confirmed a significant reduction in *LAX1* expression in *lax1*-KO plants (Figure 1B).

### 2.2. Homozygous lax1-KO Plants Show Defects in Panicle Development

Phenotypic analysis demonstrated no differences in panicle length or primary branch number between *lax1*-KO plants and non-KO controls (Figure 1C–F). However, *lax1*-KO plants exhibited significantly fewer secondary branches (Figure 1G) and grains per panicle (Figure 1H), consistent with the established role of *LAX1* in promoting branch meristem initiation [9,41]. These results validate the successful generation of the *lax1*-KO line along with a matched non-KO control for transcriptomic analysis.

### 2.3. Transcriptomic Analysis

RNA sequencing (RNA-seq) was conducted on young panicles (≤5 mm) from both *lax1*-KO and non-KO plants, employing three biological replicates per genotype. After filtering, the following clean reads were obtained: 41.23, 43.76, and 40.66 million for the lax1-KO libraries, and 42.42, 42.00, and 40.85 million for the non-KO libraries. All libraries showed Q30 scores exceeding 93.21%, indicating high sequencing quality. More than 91.10% of clean reads uniquely mapped to the rice genome using HISAT2 [42] (Table S3). Pearson correlation analysis revealed higher correlations among biological replicates within each genotype (≥0.90 for both non-KO and *lax1*-KO) [43] (Figure 2A). Principal component analysis (PCA) further confirmed clear genotype-dependent clustering, with PC1 and PC2 together explaining 71.4% of the total variance (Figure 2B). Collectively, these quality control analyses demonstrate the high reproducibility of the RNA-seq data and support the reliability of subsequent differential expression analyses.

Expression analysis detected 21,229 genes in at least one sample group (Table S4). Utilizing false discovery rate (FDR) thresholds of <0.05 and |log_2_fold change (FC)| ≥ 1, 518 DEGs were identified, with 402 upregulated and 116 downregulated in *lax1*-KO plants (Figure 2C, Table S5).

Gene Ontology (GO) annotation assigned 359, 558, and 588 gene–term annotations to the molecular function, cellular component, and biological process categories, respectively (Table S6; Figure 2D). Binding (169) and catalytic activity (141) were the predominant molecular functions. Membrane (116) and membrane part (106) were prominent among cellular components, while metabolic (143) and cellular (130) processes dominated in biological processes (Figure 2D and Figure S1A–C).

Kyoto Encyclopedia of Genes and Genomes (KEGG) annotation identified 292 DEGs, with 249 assigned to 82 pathways (Table S7). Pathways with substantial numbers of DEGs included plant–pathogen interaction (14 genes), plant hormone signal transduction (12 genes), protein processing in the endoplasmic reticulum (9 genes), galactose metabolism (9 genes), and starch and sucrose metabolism (7 genes) (Tables S8–S12; Figure 2E. Detailed enrichment bubble plots with gene ratios, adjusted *p*-values, and gene counts are provided in Figure S1D).

### 2.4. qRT-PCR Validation of RNA-seq Data

To validate the RNA-seq data, qRT-PCR was performed on 15 selected genes, including differentially expressed and non-significant genes, with nine known panicle regulators (*LAX2*, *MOC1*, *DEP1*, *DEP3*, *IPA1*, *APO2*, *RCN4*, *OsSHI1*, and *G1*) and six randomly selected genes (two upregulated, two downregulated, and two non-significant). qRT-PCR results largely aligned with RNA-seq trends, confirming the reliability of the transcriptomic data (Figure 3).

### 2.5. Loss of LAX1 Is Associated with Altered Expression of Hormone-Related and PIN Genes

Auxin-related genes constituted a significant portion of the hormone-responsive transcripts, with multiple auxin signaling components showing differential expression between the *lax1*-KO and non-KO lines (Figure 4A,B). Among these, *OsPID*, the rice ortholog of Arabidopsis PINOID kinase, was downregulated in the *lax1*-KO line (Figure 4B), consistent with the established role of *LAX1* as a transcriptional activator of *OsPID* [39]. Given that *OsPID* functions by phosphorylating *PIN* auxin efflux carriers to regulate auxin distribution [27], we next examined whether *LAX1* also affects the expression of *PIN* family members. Several *PIN* genes known to influence rice development exhibited marginal or undetectable changes in our RNA-seq data (Table S13), prompting qRT-PCR validation of their expression. qRT-PCR analysis confirmed that *OsPIN1c*, *OsPIN1d*, and *OsPIN2* were significantly downregulated in the *lax1*-KO mutant, whereas *OsPIN3t* and *OsPIN9* were upregulated. By contrast, *OsPIN1a*, *OsPIN1b*, *OsPIN5b*, and *OsPIN8* did not exhibit significant changes (Figure 4C, Table S13). Notably, among the four *OsPIN1* paralogs, only *OsPIN1c* and *OsPIN1d*—which are specifically expressed in young panicles—showed significant downregulation, while *OsPIN1a* and *OsPIN1b* remained unchanged.

Additionally, altered expression was also observed for several other auxin-related genes. *OsGH3.1* and *OsGH3.3* were downregulated in the *lax1*-KO line (Figure 4A,B), which is consistent with previous reports [39]. *OsIAA14*, *OsIAA23*, and *OsIAA24* also exhibited significant changes (Figure 4A,B). *OsIAA7* showed a trend toward downregulation (log_2_FC = −0.896) but did not meet our stringent threshold; however, qRT-PCR confirmed its significant downregulation (FC = 0.47, *p* = 0.000019). Beyond auxin, we also detected altered expression of genes involved in other hormone pathways. Specifically, the ABA-related genes *OsPP2C09* and *OsPP2C49*, the cytokinin response regulators *OsRR3* and *OsRR9*, and the gibberellin-induced gene *OsGASR9* all showed significant expression changes in the *lax1*-KO mutant (Figure 4A,B).

### 2.6. Loss of LAX1 Is Associated with Altered Expression of Starch and Sucrose Metabolism Genes

KEGG pathway analysis revealed that starch and sucrose metabolism was among the substantially enriched pathways among the downregulated DEGs (Figure 2E). We therefore examined the expression of genes involved in this pathway in more detail. Multiple genes associated with starch and sucrose metabolism were downregulated in the *lax1-KO* line, including *OsTPP4*, *OsTPP1*, *OsTPP9*, *OsHXK7*, *OsTPS8*, and several glucan endo-1,3-beta-glucosidase genes (Figure 5). Notably, *OsTPP4* and *OsHXK7* were consistently downregulated across this study and previous reports [33,39].

### 2.7. Altered Expression of Known Panicle Regulators in the lax1-KO Mutant

To assess whether known panicle development regulators are affected by *LAX1* disruption, we examined the expression of 41 previously characterized panicle-related genes in our RNA-seq dataset (Table 1). Among these, 8 genes (*IPA1*, *OsSHI1*, *LAX1*, *DEP1*, *DEP3*, *OsTB1*, *G1*, and *APO2/RFL*) showed differential expression in the *lax1*-KO line, with qRT-PCR validation confirming the downregulation of *IPA1* and *OsTB1* and the upregulation of *DEP1* and *DEP3* (Figure 3 and Figure 6A). Additionally, both *OsCUC1* and *OsCUC3* were significantly downregulated (Figure 6B,C), and several MADS-box genes, including *OsMADS4*, *OsMADS51*, and *OsMADS56*, also exhibited reduced expression (Figure 6D–F). *APO2/RFL* was downregulated in the *lax1*-KO line (Figure 3), as were *FZP* and *APO1* (Figure 6G,H). By contrast, the expression levels of *OsbHLH067*, *OsbHLH068*, *OsbHLH069*, and *LAX2* did not change significantly in the *lax1*-KO mutant (Figure 3 and Figure 6I–K).

### 2.8. Enrichment of Transcription Factor-Binding Motifs in DEG Promoters

To identify potential transcriptional regulators cooperating with *LAX1*, we performed motif enrichment analysis on the promoters of the 518 DEGs. A total of 173 motifs were significantly enriched among the 763 plant TF-binding motifs tested (adjusted *p* < 0.05, log_2_ odds ratio > 0). The most significantly enriched motifs included CAMTA2 (MA0969.2; adjusted *p* = 3.56 × 10^−15^), CAMTA3 (MA0970.2; adjusted *p* = 8.51 × 10^−14^), FAR1 (MA1382.2; adjusted *p* = 5.59 × 10^−13^), FHY3 (MA0557.2; adjusted *p* = 6.59 × 10^−13^), and CAMTA1 (MA1197.2; adjusted *p* = 1.03 × 10^−11^), which belong to the calmodulin-binding transcription activator (CAMTA) and FRS/FRF TF families. The remaining top motifs were predominantly associated with BES/BZR and bHLH families, including BZR1, TSAR2, BAM8, BMY2, bHLH78, TSAR1, ILR3, and URI (Figure 7). Pairwise similarity analysis of the top 13 PWMs revealed substantial redundancy in their DNA-binding preferences, with multiple motifs sharing high similarity (normalized similarity ≥ 0.89; Figure 7). Among the enriched motifs, the bHLH family was the most frequently represented, while the CAMTA and FRS/FRF family motifs (CAMTA1/2/3 and FAR1/FHY3) exhibited the highest significance.

## 3. Discussion

Panicle architecture is an important determinant of grain yield in rice, and understanding its regulation is of considerable interest for both basic biology and breeding. The bHLH transcription factor *LAX1* serves as a key regulator of AM formation, with its expression localized to the boundary between the initiating AM and SAM. In this study, CRISPR/Cas9 was employed to generate a *LAX1* knockout mutant, followed by transcriptome profiling of young panicles to elucidate the *LAX1*-regulated gene network during early panicle development.

Consistent with previous reports on allelic series in *lax1* mutants [9,41], the *lax1*-KO line exhibited significantly fewer secondary branches and a reduced grain number per panicle, while primary branch number and panicle length remained unaffected (Figure 1C–H). This phenotype aligns with those observed in moderate-to-strong *lax1* alleles, confirming the putative loss-of-function status of our line. The consistency of panicle defects across mutants supports the specific role of *LAX1* in panicle development, although future complementation studies would provide additional causal evidence.

The number of DEGs identified in this study (518) is smaller than the 2100 DEGs reported in a recent transcriptomic analysis of *lax1* mutants [39]. This discrepancy likely reflects our later sampling stage (≤5 mm compared to ≤2 mm), more stringent fold-change threshold (2-fold vs. 1.5-fold), different mutant alleles (a CRISPR/Cas9-induced frameshift deletion vs. a point mutation in the bHLH domain), and more conservative statistical correction (DESeq2 with FDR < 0.05 vs. Q < 0.05). To formally compare the two datasets, we performed a DEG overlap analysis using genes detected in both studies. Of the 2100 DEGs reported by Fu et al. [39], 1667 had valid LOC identifiers and were included; one of our upregulated genes lacked an LOC identifier and was excluded. Overlap analysis revealed that 131 of our 518 DEGs (25.3%) overlapped with the Fu et al. dataset, with 79 consistently upregulated and 37 consistently downregulated (direction concordance: 88.5%, 116/131; hypergeometric test *p* < 0.001; Figure S2, Table S14). Despite these differences, the core transcriptional trends—particularly OsPID downregulation and the enrichment of hormone and metabolic pathways—are consistently observed across both datasets, supporting the robustness of the core *LAX1* regulatory network. The remaining 387 genes (74.7%) represent unique candidates identified under our more stringent conditions, providing a refined set for further analysis. The convergence of these trends across two independent mutant lines and analytical thresholds provides independent corroboration of the core *LAX1*-regulated transcriptome.

A key finding of this study is the member-specific alteration of *PIN* gene expression in the *lax1*-KO mutant. Among the four *OsPIN1* paralogs, only *OsPIN1c* and *OsPIN1d*—which are specifically expressed in young panicles and act redundantly in panicle formation—were downregulated, whereas *OsPIN1a* and *OsPIN1b*—which primarily function in root and shoot development—remained unchanged (Figure 4C) [28,44,45]. This expression pattern is functionally relevant, as the *ospin1c/1d* double mutant mimics the panicle defects observed in *lax1* mutants [28]. The downregulation of *OsPIN2*, known to affect tiller number and plant height [46], may contribute to phenotypes beyond panicle architecture, while the upregulation of *OsPIN3t* and *OsPIN9* may represent compensatory responses to disrupted auxin transport, as observed in the *bos1-1* (*LAX1* allele) mutant [47]. By contrast, *OsPIN5b* and *OsPIN8* did not change significantly (Figure 4C). OsPIN5b is an ER-localized PIN protein, and its lack of alteration is consistent with its exclusion from the canonical LAX1-OsPID module that targets plasma membrane PINs; instead, it is likely regulated by independent pathways as its overexpression impairs cold tolerance through perturbing ROS homeostasis [48,49]. *OsPIN8*, another non-canonical *PIN* member, also remained unchanged, further supporting the specificity of the *LAX1*-mediated *PIN* regulation. Together with the observed physical interaction between OsPID and PIN proteins [27], these findings suggest that *LAX1* may influence auxin transport through member-specific *PIN* expression changes. However, direct evidence of altered auxin distribution or PIN protein localization is required to confirm this hypothesis. Beyond auxin, we observed altered expression of genes involved in ABA, cytokinin, and gibberellin pathways (Figure 4A,B), indicating that *LAX1* may influence multiple hormone-signaling pathways beyond auxin.

The trehalose-6-phosphate (Tre6P) pathway links sugar availability to developmental programs and plays an important role in regulating meristem activity in grasses [33]. The consistent downregulation of sugar metabolism genes—particularly *OsTPP4* and *OsHXK7*—across our study and previous reports on *lax1* and *Osbhlh067/068/069* triple mutants [33,39] points to a conserved link between *LAX1* and Tre6P signaling during panicle development (Figure 5). Variations in other affected genes among these studies may reflect differences in sampling stages or threshold settings. In our dataset, *OsTPP1* and *OsTPP9* also showed significant downregulation in the *lax1*-KO mutant, emerging as candidate components of the *LAX1*-associated Tre6P pathway. However, genetic or biochemical validation, as well as direct measurements of Tre6P and sugar metabolites, will be required to confirm their functional involvement. Moreover, because TPP enzymes catalyze Tre6P dephosphorylation, reduced TPP expression could paradoxically increase Tre6P levels rather than simply reducing pathway activity. Thus, the net physiological effect of OsTPP downregulation cannot be inferred from transcript changes alone.

In addition to hormone-related and metabolic pathways, we observed altered expression of multiple known panicle regulators in the *lax1*-KO mutant, including genes involved in meristem identity, boundary specification, and floral organ development (Table 1, Figure 3 and Figure 6). Among these, *IPA1* and *OsTB1* were downregulated, whereas *DEP1* and *DEP3* were upregulated (Figure 3 and Figure 6A). *IPA1* directly regulates both *OsTB1* and *DEP1* by binding to GTAC motifs in their promoters [50]. The coordinated downregulation of *IPA1* and *OsTB1* is consistent with this regulatory relationship, whereas the upregulation of *DEP1*—despite being an *IPA1* target—may reflect regulation by additional factors, relief of *IPA1* repression via *OsSHI1* downregulation [51], or stage-specific effects. *APO2/RFL*, which collaborates with *APO1* and directly regulates *LAX1* and *CUC* genes during AM specification [21,30], was downregulated in the *lax1*-KO line, likely as a feedback response to impaired AM initiation (Figure 3). Similarly, the reduced expression of *FZP* and *APO1* in the *lax1*-KO line (Figure 6G,H) is consistent with previous findings [47] and likely represents indirect effects of meristem failure or feedback regulation rather than direct transcriptional targets of *LAX1*. Given that *LAX1* and *FZP* operate through independent pathways [9,52], their functional interplay may extend beyond meristem initiation to later stages of floret development [53].

Beyond these signaling regulators, our transcriptomic analysis revealed that multiple transcription factor genes were differentially expressed in the *lax1*-KO mutant. The NAC family transcription factors *OsCUC1* and *OsCUC3*, which act redundantly in meristem and organ boundary specification [18], were significantly downregulated (Figure 6B,C). Fu et al. [39] showed that LAX1 ChIP-seq peaks are enriched for NAC-family motifs and that *LAX1* interacts with *OsNAC23*, suggesting a broader connection between *LAX1* and NAC family regulation that may extend to *OsCUC1/3*. Similarly, several MADS-box genes—including *OsMADS4*, *OsMADS51*, and *OsMADS56*—were significantly downregulated in the *lax1*-KO mutant (Figure 6D–F). Notably, Fu et al. [39] showed that *OsMADS51* (along with *OsMADS37* and *OsMADS18*) is a direct *LAX1* target, consistent with its downregulation in our mutant. Previous studies demonstrated that LAX1 physically interacts with OsMADS1, OsMADS6, and OsMADS7 [34], and similar MADS-bHLH synergistic regulation has been reported in Arabidopsis [54]. These expression changes occurred in young panicles during early reproductive development, coinciding with altered auxin and cytokinin signaling (Figure 4), suggesting that *LAX1* may influence early meristem identity by integrating TFs such as NAC and MADS-box with hormone signals, similarly to the stage-specific functions of other NAC factors in rice [55]. However, whether these effects are direct or indirect remains to be determined.

To explore whether the observed expression changes might involve broader transcriptional reprogramming through partner transcription factors, we performed motif enrichment analysis on the promoters of our DEGs. This identified 173 significantly enriched TF-binding motifs. Among the top 13 motifs, the bHLH family was the most frequently represented, whereas the CAMTA and FRS/FRF families—which belong to the broader GCM-domain structural class—exhibited the highest statistical significance (Figure 7). This pattern likely reflects that the bHLH family is large and diverse (>160 members in rice), resulting in a broader but less concentrated distribution of bHLH-related motifs, whereas the smaller CAMTA family (7 members) recognizes a more conserved CGCG-box motif, leading to more targeted enrichment in DEG promoters [38,56].

The enrichment of bHLH motifs is consistent with LAX1 itself being a bHLH transcription factor that directly binds target gene promoters [39]. Additionally, although LAX2, OsbHLH067, OsbHLH068, and OsbHLH069 were not differentially expressed in our dataset, they physically interact with LAX1 and function redundantly in AM formation [12,33,57], suggesting that these bHLH factors may act together with LAX1 to regulate shared target genes, which could account for the prominent representation of bHLH motifs among our enriched motifs. The even higher significance of CAMTA motifs is particularly noteworthy. CAMTA is a transcription factor family that has been implicated in development and stress responses in rice [56]. Indeed, CAMTA genes are expressed in reproductive tissues such as inflorescences and pistils, and their haplotypes are associated with grain weight traits [56]. This functional relevance supports the possibility that CAMTA motif enrichment in DEG promoters reflects a genuine regulatory role during panicle development. Fu et al. [39] established a paradigm showing that LAX1-bound regions are enriched for NAC, GATA, and C2H2 motifs, yet LAX1 does not directly bind these sequences; instead, it physically interacts with TFs from these families [39], indicating that LAX1 can achieve indirect regulation through protein–protein interactions. Notably, the overlap in transcriptomic changes between the *lax*1-KO and the Osbhlh067/068/069 triple mutant—particularly the downregulation of starch and sucrose metabolism genes—supports the notion that LAX1 and its protein partners may collaboratively influence shared downstream pathways [33]. Together, these observations raise the possibility that both bHLH and CAMTA family proteins may contribute to indirect regulatory layers downstream of LAX1.

Overall, these observations suggest a model in which LAX1 may function at a high hierarchical level, potentially influencing multiple functional classes of panicle genes—from meristem initiation to branching and spikelet transition—through altered expression of genes involved in auxin transport, sugar metabolism, and hormone signaling. Within this framework, LAX1 appears to operate through a multi-layered transcriptional architecture: a direct tier mediated by its own bHLH domain, and an indirect tier mediated through cooperation with other TF families, potentially including bHLH and CAMTA partners. This model is consistent with the cooperative binding framework, where transcription factors achieve regulatory specificity through cooperative interactions and multi-protein assembly on DNA, as reviewed by Slattery et al. [58] and Morgunova & Taipale [59]. However, the indirect regulatory relationships inferred from motif enrichment remain hypothetical; direct evidence—such as ChIP-qPCR for candidate partner TFs, yeast two-hybrid assays, and genetic epistasis experiments—will be required to establish causal regulatory connections.

## 4. Materials and Methods

### 4.1. Plant Materials and Growth Conditions

The japonica rice (*Oryza sativa* L. japonica) variety Zhonghua 11 (ZH11) served as the genetic background for all transgenic lines. A single homozygous *lax1*-KO line (carrying a 1-bp C deletion at position 51 bp) and its corresponding non-KO segregant were used in this study, with three biological replicates per genotype for RNA-seq and qRT-PCR analyses. Both the *lax1*-KO and non-KO lines were derived from the same T_0_ heterozygous parent. The T_2_ homozygous generation was used for all phenotypic and transcriptomic analyses.

For phenotypic characterization, plants were cultivated in the experimental field at Guangxi University (Nanning, Guangxi, China; 22°50′ N, 108°17′ E) during the late rice season (sown in early August 2025, transplanted in late August). A randomized plot design was used, with three rows per genotype and 30 plants per row at a spacing of 20 cm × 20 cm (one seedling per hill). Border rows were included to eliminate edge effects, and plants for phenotypic measurement were randomly sampled from the middle rows. For phenotypic measurements, *n* = 30 represents 30 individual plants per genotype, with one panicle collected from each plant. Grain number per panicle was defined as the total number of filled grains.

### 4.2. Generation of LAX1 Knockout Lines

CRISPR/Cas9-mediated genome editing was employed to knock out *LAX1*. The target site was designed using CRISPR-P v2.0 (http://cbi.hzau.edu.cn/crispr/). Binary constructs were prepared as previously described [60] and introduced into Agrobacterium tumefaciens strain EHA105, which was subsequently used to transform ZH11 as outlined in previous studies [61]. To assess CRISPR specificity, the top five predicted off-target loci were amplified from genomic DNA of the *lax1*-KO line and subjected to Sanger sequencing.

### 4.3. RNA Extraction, Library Construction, and Sequencing

Total RNA was extracted from young panicles (≤5 mm) of both *lax1*-KO and non-KO plants using the Spectrum^TM^ Plant Total RNA Kit (Sigma-Aldrich, St. Louis, MO, USA) following the manufacturer’s instructions. For each biological replicate, approximately 100 mg of tissue was collected from panicles pooled from about five individual plants, and all samples were harvested at the same time of day to minimize diurnal variation. Library construction and RNA-seq were conducted by Beijing BioMarker Technologies (Beijing, China). RNA concentration and integrity were evaluated using a NanoDrop 2000 spectrophotometer (Thermo Fisher Scientific, Waltham, MA, USA) and an Agilent 2100 Bioanalyzer (Agilent Technologies, Santa Clara, CA, USA) with the RNA Nano 6000 Assay Kit (Agilent Technologies, Santa Clara, CA, USA), respectively. Sequencing libraries were generated with the NEB Next Ultra^TM^ RNA Library Prep Kit for Illumina (New England Biolabs, Ipswich, MA, USA) and sequenced on an Illumina HiSeq^TM^ 2500 platform (Illumina, San Diego, CA, USA; paired-end 150 bp), yielding approximately 20 million read pairs (approximately 40 million individual reads) per sample. Three biological replicates were sequenced for each genotype.

### 4.4. RNA-seq Data Analysis

Raw reads (fastq format) were processed through in-house Perl scripts to remove reads containing adapters, reads containing poly-N, and low-quality reads, yielding clean reads (Q20 ≥ 97%, Q30 ≥ 93%). Clean reads were aligned to the MSU_7.0 reference genome (http://rice.uga.edu/) using HISAT2 with default parameters [42]. Reads containing more than one mismatch were excluded. FPKM (Fragments Per Kilobase of transcript per Million fragments mapped) value was applied to measure the expression level of a gene or transcript by StringTie using maximum flow algorithm [62]. To assess sample similarity and reproducibility, Pearson correlation analysis was performed on log_2_-transformed FPKM values using the plotCorrelation function of deepTools2 [43]. Additionally, principal component analysis (PCA) was conducted on variance-stabilizing transformation (VST)-normalized count data using ClustVis [63] with default parameters to visualize overall sample clustering and detect potential outliers.

Raw read counts were obtained from the sequencing provider and used for differential expression analysis with DESeq2 [64]. Genes with |log_2_FC| ≥ 1 and FDR < 0.05 (*p*-values corrected by Benjamini–Hochberg correction) were considered differentially expressed. GO (Gene Ontology) enrichment analysis of the DEGs was performed using the goseq R package (version 1.38.0) [65], which employs a Wallenius non-central hypergeometric distribution to correct for gene length bias. KEGG pathway enrichment analysis was performed using KOBAS [66] with Fisher’s exact test, and Benjamini–Hochberg correction was applied for multiple testing.

### 4.5. qRT-PCR

qRT-PCR was performed using the same RNA samples as those used for RNA-seq. For reverse transcription, 1 μg of total RNA was utilized with the PrimeScriptTM RT Reagent Kit containing gDNA Eraser (Takara Bio Inc., Kusatsu, Shiga, Japan). qRT-PCR was conducted with TB Green Premix Ex Taq II (Takara Bio Inc., Kusatsu, Shiga, Japan) on a LightCycler^®^ 480II (Roche Diagnostics, Basel, Switzerland). Rice Ubiquitin (LOC_Os03g13170) served as the internal control. Relative expression was determined using the 2^−ΔΔCt^ method [67]. All primer pairs were validated with amplification efficiencies of 95–105% (R^2^ > 0.99), and melting curve analysis confirmed single specific amplicons for each primer pair. Each qRT-PCR reaction included three biological replicates per genotype and three technical replicates per biological replicate. Technical replicates were averaged before statistical analysis. Statistical testing was performed on ΔCt values (normalized to Ubiquitin) between the two genotypes. Statistical significance for each gene was assessed using Student’s *t*-test between the two genotypes (KO vs. non-KO), with Benjamini–Hochberg FDR correction applied for multiple testing. Adjusted *p* < 0.05 was considered statistically significant. qRT-PCR primers are provided in Table S15.

### 4.6. Motif Enrichment Analysis

Motif enrichment analysis was performed on the promoters of DEGs identified in the *lax1*-KO versus non-KO comparison. Promoter sequences were defined as the 2000-bp region immediately upstream of the transcription start site (TSS) for each protein-coding gene based on the MSU7 rice genome annotation. The foreground set consisted of the 518 DEGs (FDR < 0.05, |log_2_FC| ≥ 1). The background set was constructed by randomly selecting the same number of genes from the remaining non-DEGs (padj > 0.05, |log_2_FC| < 0.32), following the criteria established by Oshchepkov et al. [68].

Motif enrichment was evaluated using ESDEG (https://github.com/ubercomrade/esdeg, accessed on 2 September 2026). A total of 763 plant TF-binding motifs from the JASPAR 2024 CORE Plants collection [69] were examined. Motif occurrences were evaluated across 20 recognition thresholds calibrated according to the expected recognition rate. For each motif and threshold, the observed site frequency in DEG promoters was compared with the distribution obtained from 1000 Monte Carlo background samples matched for promoter GC content. Threshold-specific probabilities were combined using the Bonferroni procedure, and resulting motif-level *p* values were corrected using the Benjamini–Hochberg procedure. Motifs with adjusted *p* < 0.05 and positive log_2_ odds ratio were considered significantly enriched.

Pairwise similarity among the top-ranking enriched motifs was calculated using a Jensen–Shannon divergence-based measure on the corresponding position weight matrices (PWMs). Motif pairs with a normalized similarity score ≥ 0.62 were visualized in a similarity matrix.

## 5. Conclusions

In summary, this study provides independent validation and a refined view of the LAX1-regulated transcriptional network in rice panicle development. The identification of 518 DEGs revealed expression changes across multiple hormone-signaling associated pathways, especially a member-specific alteration of PIN gene expression. Candidate involvement in Tre6P signaling was also observed through downregulation of *OsTPP* genes, including *OsTPP1* and *OsTPP9*, along with expression changes in meristem identity genes such as *IPA1*, *OsTB1*, *DEP1*, *DEP3*, *CUC*, and MADS-box genes.

Motif enrichment analysis further identified bHLH motifs as the most frequently represented, whereas CAMTA and FRS/FRF motifs exhibited the highest statistical significance in DEG promoters, consistent with a possible multi-layered transcriptional architecture involving both direct LAX1-mediated regulation and indirect regulation through other TF families. However, the indirect regulatory relationships inferred from motif enrichment remain hypothetical and require direct experimental validation. Collectively, these findings suggest that *LAX1* may function as an important regulator coordinating multiple aspects of panicle development through multi-layered transcriptional cascades, providing a foundation for future functional studies aimed at dissecting the combinatorial regulatory code underlying panicle architecture.

## Figures and Tables

**Figure 1:**
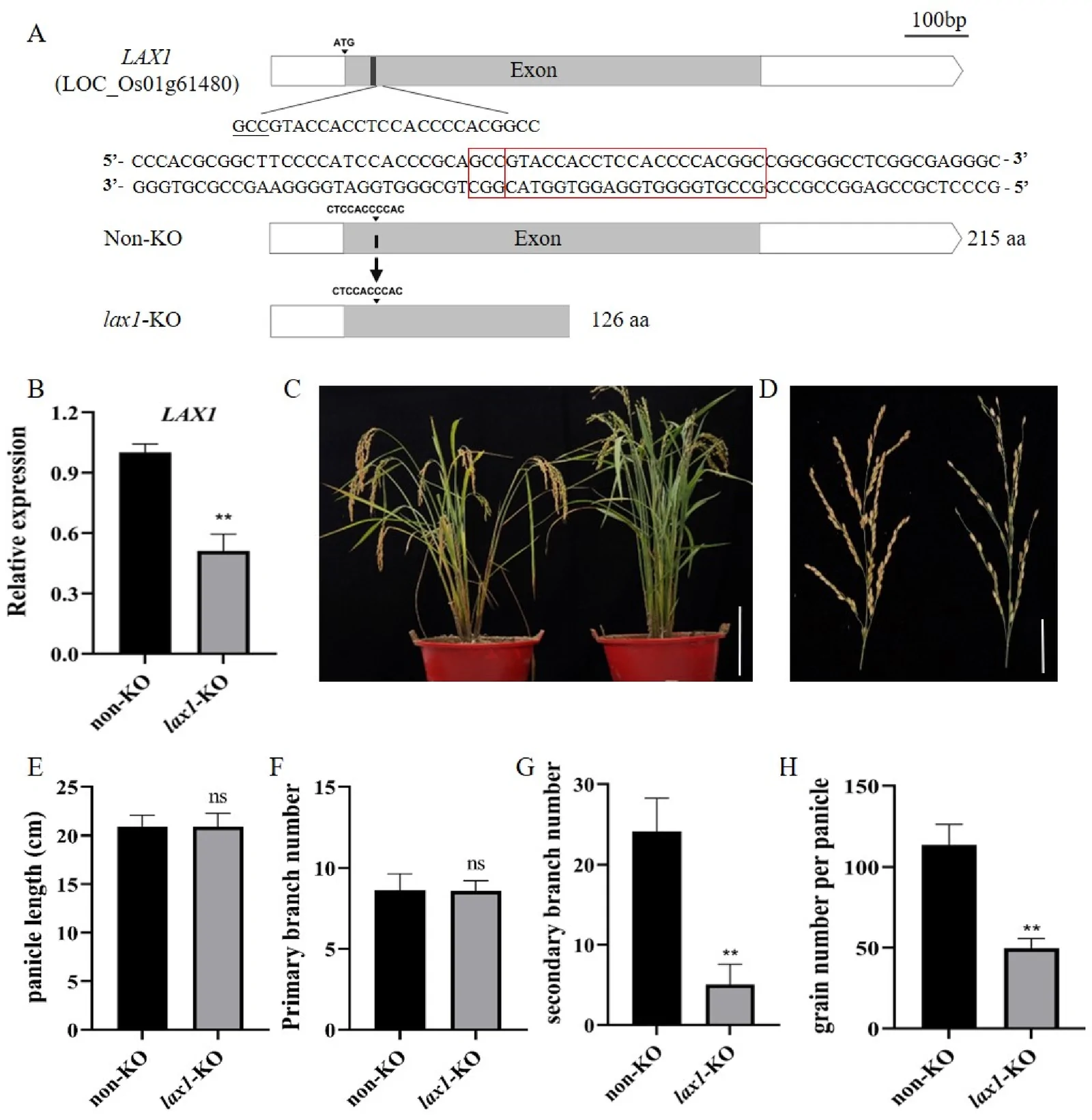
Generation and phenotypic characterization of the *lax1*-KO mutant. (**A**) Gene structure with the CRISPR/Cas9 target site of *LAX1*. Gray boxes indicate exons, and white boxes indicate untranslated regions. The protospacer-adjacent motif (PAM) sequence is underlined. Details of the target site and adjacent sequences are shown below. (**B**) Relative *LAX1* expression in KO and non-KO lines measured by qRT-PCR. Values are means ± SD. (*n* = 3). ** represents *p* < 0.01 (Student’s *t*-test). (**C**,**D**) Whole-plant (**C**) and panicle (**D**) phenotypes of non-KO and *lax1*-KO plants. Scale bars are 5 cm in (**C**) and 2 cm in (**D**). (**E**–**H**) Quantification of panicle length (**E**), primary branch number (**F**), secondary branch number (G), and grain number per panicle (filled grains) (**H**). Data are presented as means ± SD (*n* = 30). ** *p* < 0.01 (Student’s *t*-test). ns, not significant.

**Figure 2:**
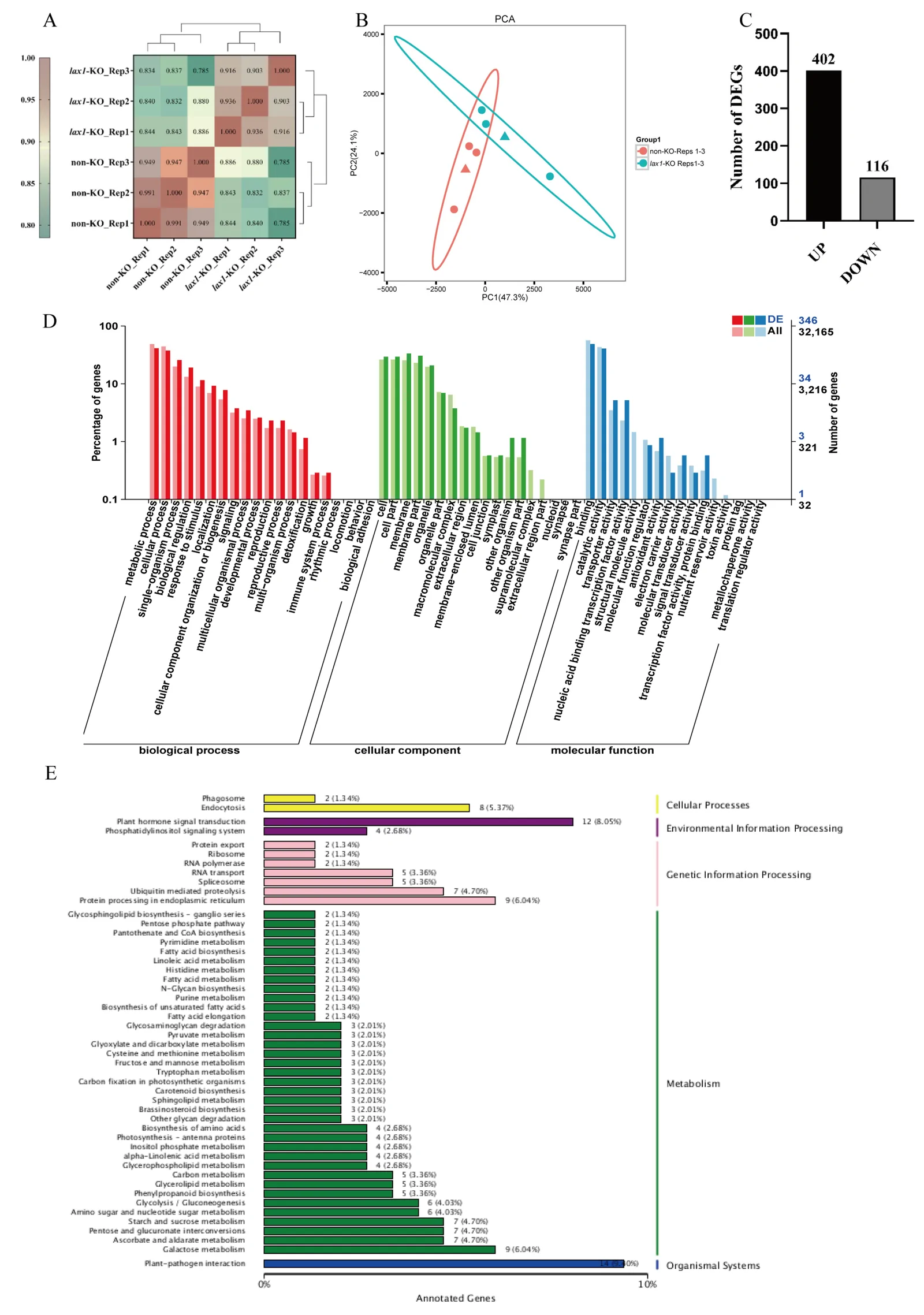
Overview of RNA-seq analysis. (**A**) Pairwise correlation analysis (Pearson’s correlation coefficient) among all samples. Color scale indicates correlation coefficients ranging from low (green) to high (red). (**B**) PCA plot of all samples. PC1 and PC2 explain 47.3% and 24.1% of the total variance, respectively. Red and cyan indicate non-KO and *lax1*-KO, respectively. Circles represent individual biological replicates, and triangles represent group centroids. (**C**) Number of diffrentially expressed genes. (**D**) Gene Ontology enrichment analysis. The vertical axis shows the top enriched GO terms, and the horizontal axis shows the number of DEGs annotated to each term. (**E**) KEGG pathway enrichment analysis. The vertical axis shows the top 50 enriched pathways, and the horizontal axis shows the number of DEGs in each pathway.

**Figure 3:**
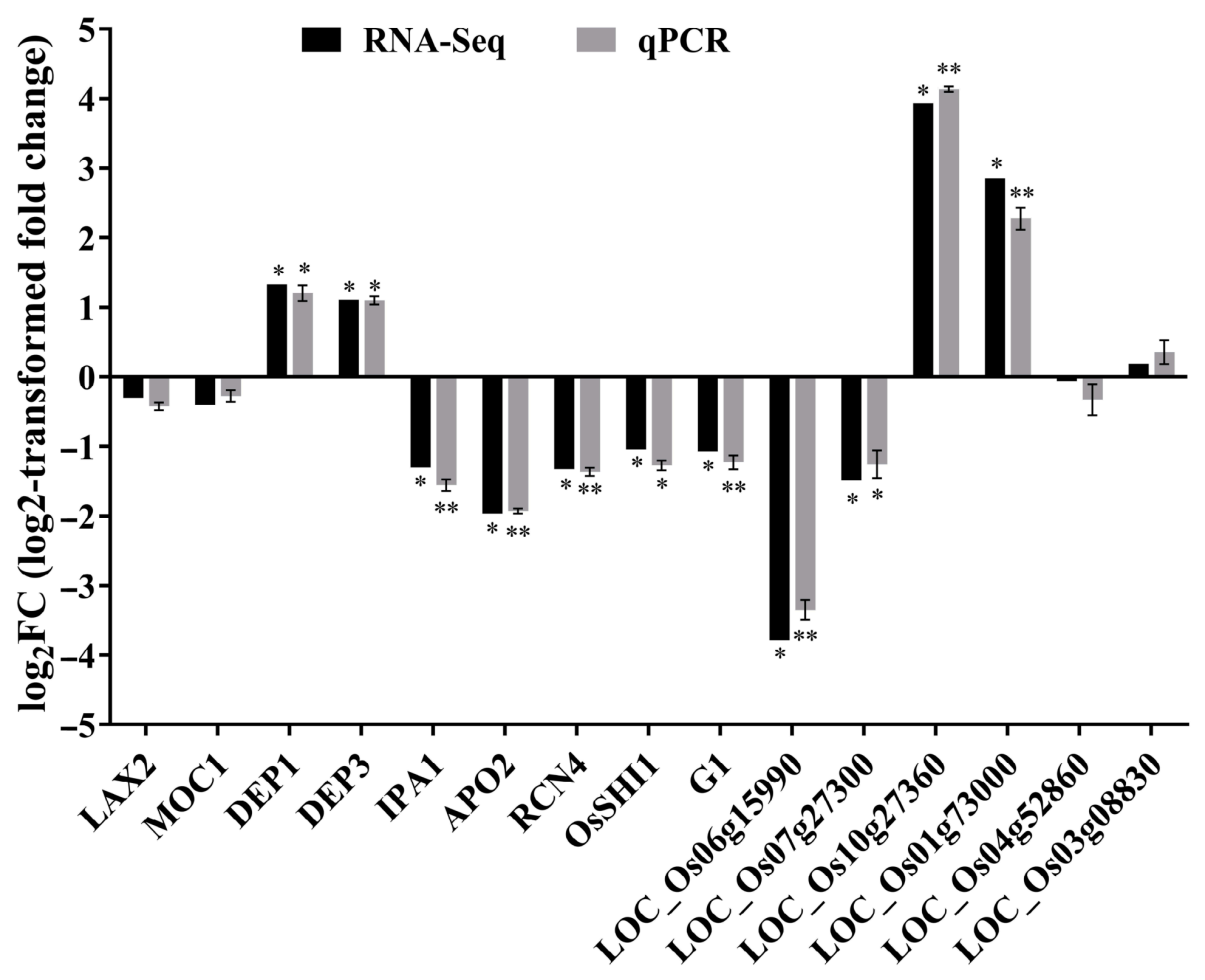
qRT-PCR validation of RNA-seq data for selected genes in *lax1*-KO versus non-KO panicles. Expression levels of 15 selected genes were measured by qRT-PCR, including known panicle regulators (*LAX2*, *MOC1*, *DEP1*, *DEP3*, *IPA1*, *APO2*, *RCN4*, *OsSHI1*, *G1*) and randomly selected genes. For qRT-PCR, data are presented as means ± SD (*n* = 3), * *p* < 0.05; ** *p* < 0.01 (Student’s *t*-test). For RNA-seq, * |log_2_FC| ≥ 1, FDR < 0.05. The y-axis represents log2-transformed fold changes (log_2_FC).

**Figure 4:**
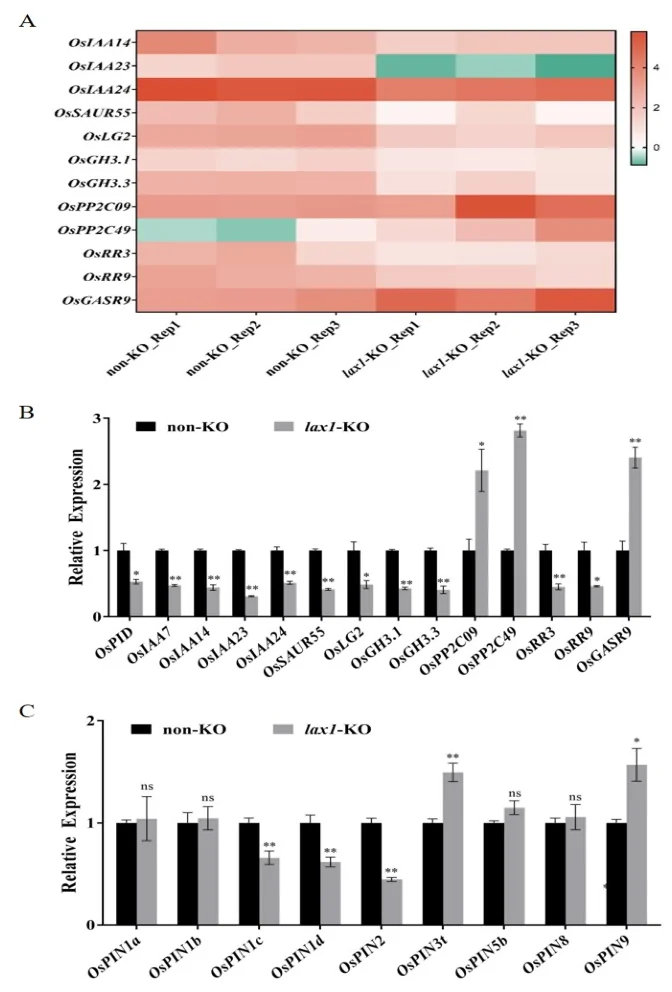
Expression profiles of plant hormone signal transduction-related genes. (**A**) Heatmap of hormone-related DEGs. Red and green indicate high and low expression, respectively. Expression values are shown as FPKM (Fragments Per Kilobase of transcript per Million fragments mapped) with a log_2_ scale. (**B**) qRT-PCR validation of hormone-related DEGs. (**C**) qRT-PCR validation of PIN family members. Data are presented as means ± SD (*n* = 3). * *p* < 0.05; ** *p* < 0.01; ns, not significant (Student’s *t*-test).

**Figure 5:**
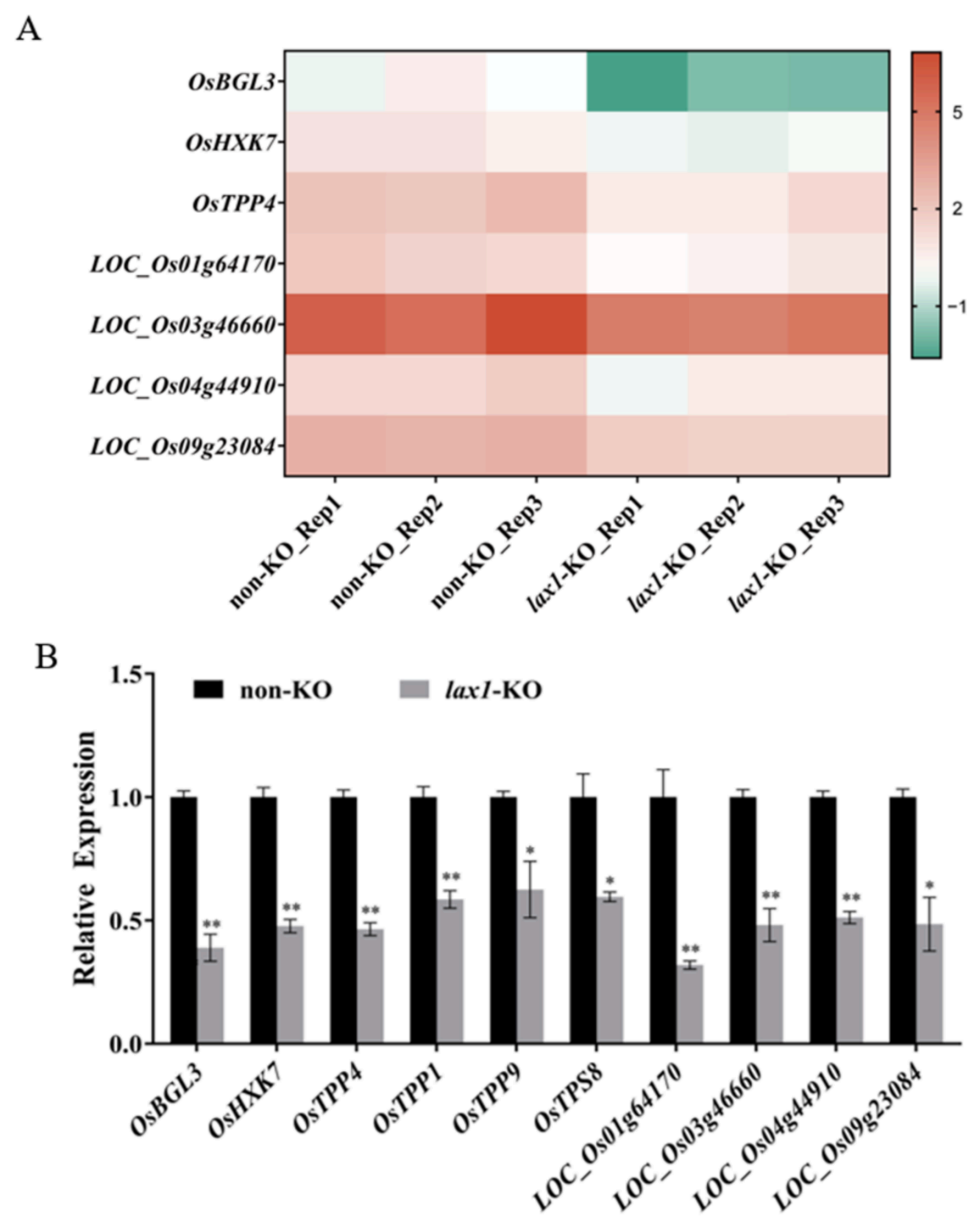
Expression profiles of starch- and sucrose metabolism-related genes. (**A**) Heatmap showing the expression patterns of starch- and sucrose metabolism-related DEGs. Red and green indicate high and low expression levels, respectively. Expression values are shown as FPKM with a log_2_ scale. (**B**) qRT-PCR validation of starch- and sucrose metabolism-related DEGs. Data are presented as means ± SD (*n* = 3). * *p* < 0.05; ** *p* < 0.01 (Student’s *t*-test).

**Figure 6:**
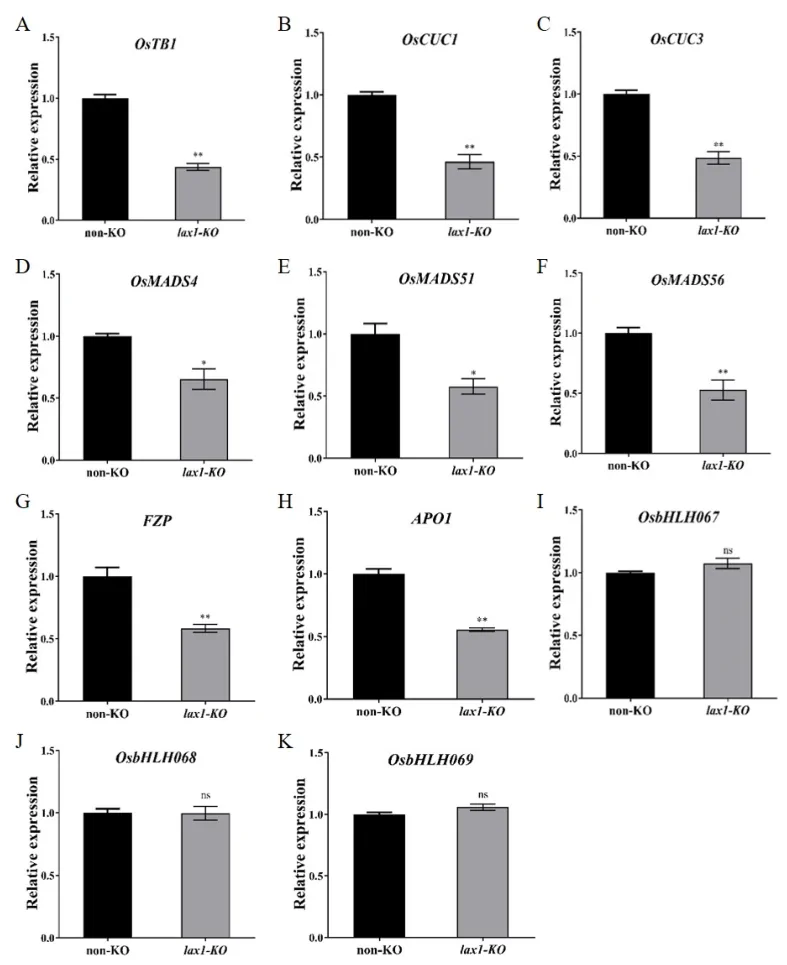
qRT-PCR validation of genes involved in panicle development. Relative expression levels of *OsTB1* (**A**); *OsCUC1* and *OsCUC3* (**B**,**C**); *OsMADS4*, *OsMADS51*, and *OsMADS56* (**D**–**F**); *FZP* and *APO1* (**G**,**H**); and *OsbHLH067*, *OsbHLH068*, and *OsbHLH069* (**I**–**K**) in non-KO and *lax1*-KO mutants. Data are presented as means ± SD (*n* = 3), * *p* < 0.05; ** *p* < 0.01, ns, not significant (Student’s *t*-test).

**Figure 7:**
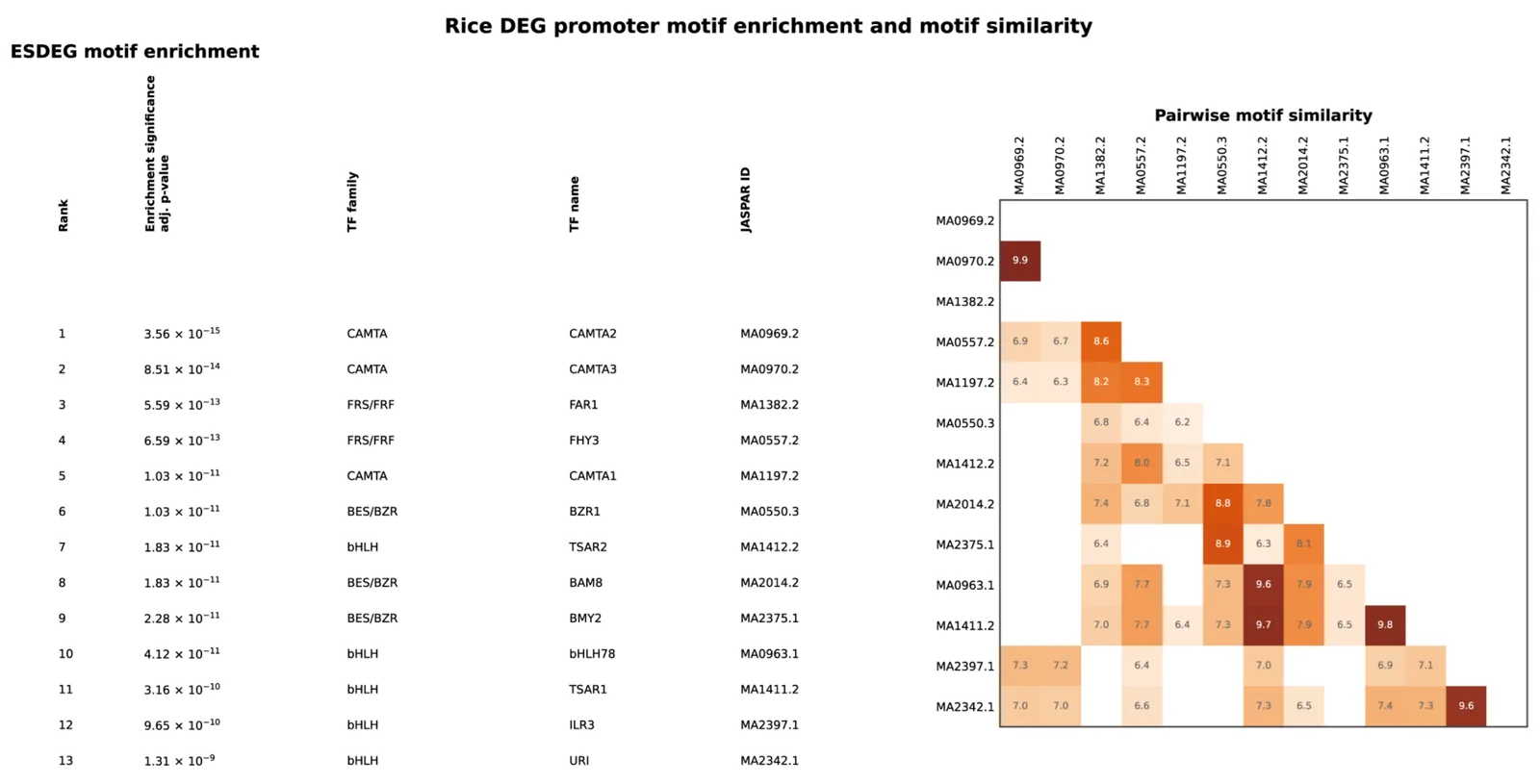
Ranking, classification, and pairwise similarity of transcription factor-binding motifs enriched in the promoters of DEGs. Top 13 significantly enriched motifs (Benjamini–Hochberg-adjusted *p* < 0.05), with TF family, TF name, and JASPAR ID. Pairwise similarity matrix of the corresponding position weight matrices (PWMs). Similarity was calculated using optimal ungapped alignment in both forward- and reverse-complement orientations. Colored cells indicate normalized similarity ≥ 0.62; color intensity increases with similarity. Values represent similarity scores ×10. TF names and classifications are based on JASPAR annotations and indicate similarity in DNA-binding preference, not direct binding by the corresponding rice TFs. Motif enrichment was performed using ESDEG with the JASPAR 2024 CORE Plants database (see Section 4.6).

**Table 1:** The expression changes in panicle architecture-related genes in lax1-KO.

Function	Gene Symbol	Accession Number	FDR	log_2_FC
Primary branch	*LOG*	LOC_Os01g40630	0.99436126	0.00436740
	*DST*	LOC_Os03g57240	0.70480669	−0.22481724
	***IPA1/OsSPL14***	**LOC_Os08g39890**	**0.00304842**	**−** **1.30190882**
	*Gn1a*	LOC_Os01g10110	ND	ND
	***OsSHI1***	**LOC_Os09g36160**	**0.02469950**	**−** **1.04709989**
	*OsOTUB1*	LOC_Os08g42540	0.95854797	0.02636662
	*OsSPL7*	LOC_Os04g46580	0.93258635	0.07546396
	*OsSPL17*	LOC_Os09g31438	0.14513359	0.68078470
	*OsNAC2*	LOC_Os04g38720	0.72155582	−0.255555553
	*FON2/4*	LOC_Os11g38270	ND	ND
	*FON1*	LOC_Os06g50340	0.61691098	−0.25884492
Secondary branch	*FZP*	LOC_Os07g47330	ND	ND
	*OsMFT1*	LOC_Os06g30370	ND	ND
	*NAL1/SPIKE*	LOC_Os04g52479	0.05782987	0.29941775
	*GNP1*	LOC_Os03g63970	0.58196191	0.32584565
	*TAW1*	LOC_Os10g33780	0.63916248	0.39318311
	*GN2*	LOC_Os02g56630	ND	ND
	*OsGRF6*	LOC_Os03g51970	0.19086096	−0.31591925
	*GSN1*	LOC_Os05g02500	0.73710512	0.12247740
	*GLW7*	LOC_Os07g32170	0.24174398	−0.23658485
	*NOG1*	LOC_Os01g54860	0.56769440	0.20071352
Lateral spikelets	***LAX1***	**LOC_Os01g61480**	**0.04011291**	**−** **1.56610054**
	*LAX2/GNP4*	LOC_Os04g32510	0.67687271	−0.30438598
	*MOC1/GNP6*	LOC_Os06g40780	0.54335591	−0.40547997
Multifloret spikelet	***G1***	**LOC_Os07g04670**	**0.00698272**	**−** **1.07132537**
	*EG1*	LOC_Os01g67430	ND	ND
	*PAP2/OsMADS34*	LOC_Os03g54170	0.59020330	0.20989201
	*MFS1*	LOC_Os05g41760	0.29727261	0.69571772
	*SNB/SSH1*	LOC_Os07g13170	0.25979082	−0.25038011
	*OsIDS/AP2-2*	LOC_Os03g60430	0.12286515	−0.35750086
	*LF1*	LOC_Os03g01890	0.51445645	−0.13890717
Panicle type	***DEP1***	**LOC_Os09g26999**	**0.00762794**	**1.33095445**
	*SPED1*	LOC_Os06g39650	0.97291436	0.02175464
	*APO1*	LOC_Os06g45460	ND	ND
	***APO2/RFL***	**LOC_Os04g51000**	**0.00860021**	**−** **1.96337841**
	*SP1*	LOC_Os11g12740	0.90014610	0.09597448
	*ASP1/OsTPR2*	LOC_Os08g06480	0.55746124	−0.13493866
	*EP2/DEP2*	LOC_Os07g42410	0.82837548	0.06275634
	*EP3/LP*	LOC_Os02g15950	0.70312365	−0.25590298
	***DEP3***	**LOC_Os06g46350**	**0.00143445**	**1.10760890**
	***OsTB1/FC1***	**LOC_Os03g49880**	**0.02388771**	**−** **1.14151000**

ND, not detected in the RNA-seq data. Genes highlighted in bold are RNA-seq DEGs meeting the significance criteria (FDR < 0.05, |log_2_FC|≥ 1).

## Data Availability

All data supporting the results and conclusions are included in the article and additional files. The raw transcriptome data have been deposited in the Genome Sequence Archive (GSA) at the National Genomics Data Center (CNCB) under BioProject PRJCA010330 and are publicly accessible at https://ngdc.cncb.ac.cn/gsa (accessed on 1 July 2023).

## Supplementary Materials

The following supporting information can be downloaded at https://www.mdpi.com/article/10.3390/plants15192923/s1, Table S1: The target site sequence of the 19 T_0_ transgenic plants; Table S2: Predicted top five off-target sites and sequencing validation in the *lax1*-KO line; Table S3: Statistical analysis of transcriptome sequencing data; Table S4: Expression profiles of all genes; Table S5: Differentially expressed genes; Table S6: Genes assigned to GO; Table S7: Genes assigned to KEGG pathway; Table S8: Genes assigned to plant-pathogen interaction; Table S9: Genes assigned to plant hormone signal transduction pathway; Table S10: Genes assigned to protein processing in endoplasmic reticulum; Table S11: Genes assigned to galactose metabolism; Table S12: Genes assigned to starch and sucrose metabolism; Table S13: The expression changes in *PIN* family genes implicated in rice development in *lax1*-KO; Table S14: Overlapping DEGs between this study and Fu et al. (2026) [39]; Table S15: The qPCR primers used in this study; Figure S1: GO and KEGG enrichment bubble plots of DEGs; Figure S2: Venn diagram showing the overlap of DEGs between this study and Fu et al. (2026) [39].

## Abbreviations

The following abbreviations are used in this manuscript:

AM	Axillary meristem
SAM	Shoot apical meristem
IM	Inflorescence meristem
bHLH	Basic/helix–loop–helix
RNA-seq	RNA sequencing
qRT-PCR	Quantitative reverse-transcription PCR
GO	Gene Ontology
KEGG	Kyoto Encyclopedia of Genes and Genomes
