# Supplementary material for: Independent Validation and Refinement of the LAX1-Regulated Transcriptional Network in Rice Panicle Development
Source: Plants (Basel). 2026 Sep 24;15(19):2923. doi: 10.3390/plants15192923 (PMC13666667; doi:10.3390/plants15192923)
Supplement: Supplementary file 1 [file plants-15-02923-s001.zip › Table S1. The target site sequence of the 19 T0 transgenic plants.pdf]

Table S1. The target site sequence of the 19 T_0 transgenic plants

Wild-type	ACCCGCAGCCGTACCACCTCCACCCC-	
LAX1^{CRISP}-1	ACCCGCAGCCGTACCACCTCCACCCC**T**ACGGCC	+1
	ACCCGCAGCCGTACCACCTCCACCCC**C**ACGGCC	+1
LAX1^{CRISP}-2	ACCCGCAGCCGTACCACCTCCACCC--ACGGCC	-1
	ACCCGCAGCCGTACCACCTCCACCCC**T**ACGGCC	+1
LAX1^{CRISP}-3	ACCCGCAGCCGTACCACCTCCACCC--ACGGCC	-1
	ACCCGCAGCCGTACCACCTCCACCC--ACGGCC	-1
LAX1^{CRISP}-4	ACCCGCAGCCGTACCACCTCCACCC--ACGGCC	-1
	ACCCGCAGCCGTACCACCTCCACCCC-ACGGCC	0
LAX1^{CRISP}-5	ACCCGCAGCCGTACCACCTCCACCCC-ACGGCC	0
	ACCCGCAGCCGTACCACCTCCACCCC-ACGGCC	0
LAX1^{CRISP}-6	ACCCGCAGCCGTACCACCTCCACCCC**C**ACGGCC	+1
	ACCCGCAGCCGTACCACCTCCACCCC**T**ACGGCC	+1
LAX1^{CRISP}-7	ACCCGCAGCCGTACCACCTCCACCCC**T**ACGGCC	+1
	ACCCGCAGCCGTACCACCTCCACCCC**C**ACGGCC	+1
LAX1^{CRISP}-8	ACCCGCAGCCGTACCACCTCCACCCC**C**ACGGCC	+1
	ACCCGCAGCCGTACCACCTCCACCCC-ACGGCC	0
LAX1^{CRISP}-9	ACCCGCAGCCGTACCACCTCCACCCC-ACGGCC	0
	ACCCGCAGCCGTACCACCTCCACCCC-ACGGCC	0
LAX1^{CRISP}-10	ACCCGCAGCCGTACCACCTCCACCCC-ACGGCC	0
	ACCCGCAGCCGTACCACCTCCACCCC-ACGGCC	0
LAX1^{CRISP}-11	ACCCGCAGCCGTACCACCTCCACCCC-ACGGCC	0
	ACCCGCAGCCGTACCACCTCCACCCC**C**ACGGCC	+1
LAX1^{CRISP}-12	ACCCGCAGCCGTACCACCTCCACCCC**T**ACGGCC	+1
	ACCCGCAGCCGTACCACCTCCACCCC-ACGGCC	0
LAX1^{CRISP}-13	ACCCGCAGCCGTACCACCTCCACCC--ACGGCC	-1
	ACCCGCAGCCGTACCACCTCCACCC--ACGGCC	-1
LAX1^{CRISP}-14	ACCCGCAGCCGTACCACCTCCACCCC-ACGGCC	0
	ACCCGCAGCCGTACCACCTCCACCCC-ACGGCC	0
LAX1^{CRISP}-15	ACCCGCAGCCGTACCACCTCCACCCC-ACGGCC	0
	ACCCGCAGCCGTACCACCTCCACCCC-ACGGCC	0
LAX1^{CRISP}-16	ACCCGCAGCCGTACCACCTCCACCCC**T**ACGGCC	+1
	ACCCGCAGCCGTACCACCTCCACCCC**C**ACGGCC	+1
LAX1^{CRISP}-16	ACCCGCAGCCGTACCACCTCCACCCC**T**ACGGCC	+1
	ACCCGCAGCCGTACCACCTCCACCC--ACGGCC	-1
LAX1^{CRISP}-18	ACCCGCAGCCGTACCACCTCCACCC--ACGGCC	-1
	ACCCGCAGCCGTACCACCTCCACCCC-ACGGCC	0
LAX1^{CRISP}-19	ACCCGCAGCCGTACCACCTCCACCCC-ACGGCC	0
	ACCCGCAGCCGTACCACCTCCACCCC-ACGGCC	0

The sequence of the target sites for two alleles of each transgenic plant. The mutant site are shown in bold. The numeric value on the right represents the mutant type for each allele, + represents nucleotide insertion, - represents nucleotide deletion, the number represents the insertion or deletion number of nucleotide.
