# Supplementary material for: Independent Validation and Refinement of the LAX1-Regulated Transcriptional Network in Rice Panicle Development
Source: Plants (Basel). 2026 Sep 24;15(19):2923. doi: 10.3390/plants15192923 (PMC13666667; doi:10.3390/plants15192923)
Supplement: Supplementary file 1 [file plants-15-02923-s001.zip › Table S13. The expression changes of PIN family genes implicated in rice development in lax1-KO.pdf]

Table S13. The expression changes of *PIN* family genes implicated in rice development in *lax1*-KO

Gene symbol	Accession number	FDR	log2FC
OsPIN1a	*LOC_Os06g12610*	0.30035290	-0.48958256
OsPIN1b	*LOC_Os02g50960*	0.37411761	0.36798084
OsPIN1c	*LOC_Os11g04190*	0.00143445	-0.72808594
OsPIN1d	*LOC_Os12g04000*	0.00906529	-0.66789374
OsPIN2	*LOC_Os06g44970*	ND	ND
OsPIN5b	*LOC_Os08g41720*	ND	ND
OsPIN9	*LOC_Os01g58860*	0.06090121	1.01493637
OsPIN10a/OsPIN3t	*LOC_Os01g45550*	0.02469950	0.68486880

ND not detected in the RNA-seq
