# Supplementary material for: Independent Validation and Refinement of the LAX1-Regulated Transcriptional Network in Rice Panicle Development
Source: Plants (Basel). 2026 Sep 24;15(19):2923. doi: 10.3390/plants15192923 (PMC13666667; doi:10.3390/plants15192923)
Supplement: Supplementary file 1 [file plants-15-02923-s001.zip › Table S15. The qPCR primers used in this study.pdf]

Table S15. The qPCR primers used in this study

Gene ID	Gene name	Sequence(5'->3')
LOC_Os01g61480	*LAX1*	Forward primer: TGCCTCCTTTGCCTTGCT
		Reverse primer: TGGACATTGCACACCGAGTAG
LOC_Os04g32510	*LAX2*	Forward primer:
		Reverse primer:
LOC_Os06g40780	*MOC1*	Forward primer:
		Reverse primer:
LOC_Os09g26999	*DEP1*	Forward primer: GCGACGAGCCATGCTGTAAG
		Reverse primer: AGCTTGGACAGGAGCACGAG
LOC_Os06g46350	*DEP3*	Forward primer:
		Reverse primer:
LOC_Os08g39890	*IPA1*	Forward primer: CTTCGACCAAACCAGCAACA
		Reverse primer: AGGCAGCATGAGGTTCTTCAG
LOC_Os04g51000	*APO2*	Forward primer:
		Reverse primer:
LOC_Os04g33570	*RCN4*	Forward primer: CTCCAGGGACTACTTCAGCACC
		Reverse primer: TTGGTTTTTGGCAGTTTGGTTA
LOC_Os09g36160	*OsSHI1*	Forward primer: AAGAAGGACTGCACCCACAT
		Reverse primer: GGACCCAGGTGGACTTGA
LOC_Os07g04670	*G1*	Forward primer:
		Reverse primer:
LOC_Os06g15990		Forward primer:
		Reverse primer:
LOC_Os07g27300		Forward primer:
		Reverse primer:
LOC_Os10g27360		Forward primer:
		Reverse primer:
LOC_Os01g73000		Forward primer:
		Reverse primer:
LOC_Os04g52860		Forward primer:
		Reverse primer:
LOC_Os03g08830		Forward primer:
		Reverse primer:
LOC_Os12g42020	*OsPID*	Forward primer: TACTACCTACCCGCGTCGTC
		Reverse primer: CGGTAGCTAAGGGTCAACGA
LOC_Os02g13520	*OsIAA7*	Forward primer: GGGGGATGTTTGTTTCTTCA
		Reverse primer: TGTTCTTTTCCGTCCAGAGG
LOC_Os03g58350	*OsIAA14*	Forward primer:
		Reverse primer:
LOC_Os06g39590	*OsIAA23*	Forward primer:
		Reverse primer:
LOC_Os07g08460	*OsIAA24*	Forward primer:

		Reverse primer:
LOC_Os09g37500	*OsSAUR55*	Forward primer:
		Reverse primer:
LOC_Os01g64020	*OsLG2*	Reverse primer:
		Forward primer:
LOC_Os01g57610	*OsGH3.1*	Reverse primer: GCGTCCATCAACCAGTACAA
		Forward primer: GGGCACTTGGGACTGAAGTA
LOC_Os01g12160	*OsGH3.3*	Reverse primer: ATCAGCTCAGCGTTTTCACC
		Reverse primer: TTCGTCGTCGTCTCCTCTTT
LOC_Os01g62760	*OsPP2C09*	Forward primer:
		Reverse primer:
LOC_Os05g38290	*OsPP2C49*	Forward primer:
		Reverse primer:
LOC_Os02g58350	*OsRR3*	Forward primer:
		Reverse primer:
LOC_Os11g04720	*OsRR9*	Forward primer:
		Reverse primer:
LOC_Os07g40240	*OsGASR9*	Forward primer:
		Reverse primer:
LOC_Os06g12610	*OsPIN1a*	Forward primer: GTCTGCTTCCGGTGGAACT
		Reverse primer: CGGAGAGGATGGAGATGGATT
LOC_Os02g50960	*OsPIN1b*	Forward primer: CCAGCCTCATCGGCCTCAT
		Reverse primer: CGACAGGATCGAGATGGATTTCAG
LOC_Os11g04190	*OsPIN1c*	Forward primer: CAGGCCGCTCTTCCTCAAG
		Reverse primer: TCACGGCTGTGCTCAGAATG
LOC_Os12g04000	*OsPIN1d*	Forward primer: CGTGATGACGAGGCTCATAC
		Reverse primer: CCGAGGAGGCTGGAGTAAG
LOC_Os06g44970	*OsPIN2*	Forward primer:
		Reverse primer:
LOC_Os01g45550	*OsPIN3t*	Forward primer:
		Reverse primer:
LOC_Os08g41720	*OsPIN5b*	Forward primer:
		Reverse primer:
LOC_Os01g51780	*OsPIN8*	Forward primer:
		Reverse primer:
LOC_Os01g58860	*OsPIN9*	Forward primer:
		Reverse primer:
LOC_Os07g35480	*OsBGL3*	Forward primer:
		Reverse primer:
LOC_Os05g09500	*OsHXK7*	Forward primer: AGAATTGTGGCTGAAAAACTTG
		Reverse primer: TGATCTTCGGGTGACGATGTC
LOC_Os02g51680	*OsTPP4*	Forward primer: CACCAACGCCTCCTACTCTCT
		Reverse primer: GACGACTTCCGCTTCCACTCC

LOC_Os02g44230	*OsTPP1*	Forward primer:
		Reverse primer:
LOC_Os03g26910	*OsTPP9*	Forward primer: GGGGCTGGAGGAGCTCTACTA
		Reverse primer: ATCATCGGCAGGAACTCCCTC
LOC_Os08g34580	*OsTPS8*	Forward primer: GGATGAACTGGCTGAATGG
		Reverse primer: TGCCAAGAGAATAGGGAGG
LOC_Os01g64170		Forward primer:
		Reverse primer:
LOC_Os03g46660		Forward primer:
		Reverse primer:
LOC_Os04g44910		Forward primer:
		Reverse primer:
LOC_Os09g23084		Forward primer:
		Reverse primer:
LOC_Os03g49880	*OsTB1*	Forward primer: ATCAGCAGGATCAGTGGGAG
		Reverse primer: AGCTGCAGGAGTTCGATCTT
LOC_Os06g23650	*OsCUC1*	Forward primer:
		Reverse primer:
LOC_Os08g40030	*OsCUC3*	Forward primer:
		Reverse primer:
LOC_Os05g34940	*OsMADS4*	Forward primer:
		Reverse primer:
LOC_Os01g69850	*OsMADS51*	Forward primer:
		Reverse primer:
LOC_Os10g39130	*OsMADS56*	Forward primer:
		Reverse primer:
LOC_Os07g47330	*FZP*	Forward primer:
		Reverse primer:
LOC_Os06g45460	*APO1*	Forward primer:
		Reverse primer:
LOC_Os05g42180	*OsbHLH067*	Forward primer: AGCGAAGAAACCACGGATCG
		Reverse primer: CAAAAGGCGAGACGAGTTGC
LOC_Os04g53990	*OsbHLH068*	Forward primer: CCGAGACTCGAGACACCGTC
		Reverse primer: CCGATGCGGTATCCGTCTTT
LOC_Os01g57580	*OsbHLH069*	Forward primer:
		Reverse primer:
LOC_Os03g1317	*Ubiquitin*	Forward primer: CTGTCAACTGCCGCAAGAAG
		Reverse primer: GGCGAGTGACGCTCTAGTTC
