# Supplementary material for: Independent Validation and Refinement of the LAX1-Regulated Transcriptional Network in Rice Panicle Development
Source: Plants (Basel). 2026 Sep 24;15(19):2923. doi: 10.3390/plants15192923 (PMC13666667; doi:10.3390/plants15192923)
Supplement: Supplementary file 1 [file plants-15-02923-s001.zip › Table S2. Predicted top five off-target sites and sequencing validation in the lax1-KO line.pdf]

Table S2. **Predicted top five off-target sites and sequencing validation in the *lax1*-KO line.**

Sequence	Off-score	MMs	Locus	Gene	Region	Sanger result
CCAGC**GT**GCCGACGTCGCCG**CGG**	0.580	2MMs	Chr7:-18807557	LOC_Os07g31660	CDS	No mutation
C**G**AGC**G**CGCCGACGTCGCC**A****AGG**	0.466	3MMs	Chr2:+344805	LOC_Os02g01590	CDS	No mutation
**A**CAGC**T**CGCCGACGTCG**T**CG**AGG**	0.459	3MMs	Chr7:-9038825	LOC_Os07g15600	CDS	No mutation
**G**CAG**G**ACGCCGAC**A**TCGCCG**CGG**	0.411	3MMs	Chr6:+17196888	LOC_Os06g29844	CDS	No mutation
**GG**AGCACG**T**CG**G**CGTCGCCG**AGG**	0.401	4MMs	Chr2:+5167477	LOC_Os02g09940	CDS	No mutation
