# Supplementary material for: Independent Validation and Refinement of the LAX1-Regulated Transcriptional Network in Rice Panicle Development
Source: Plants (Basel). 2026 Sep 24;15(19):2923. doi: 10.3390/plants15192923 (PMC13666667; doi:10.3390/plants15192923)
Supplement: Supplementary file 1 [file plants-15-02923-s001.zip › Table S3. Statistical analysis of transcriptome sequencing data.pdf]

Table S3. Statistical analysis of transcriptome sequencing data

Sample	Total Reads	Mapped Reads	Uniq Mapped Reads	GC Content	%≥Q30
non-KO_Rep1	42,415,840	40,701,974 (95.96%)	39,563,853 (93.28%)	51.93%	94.38%
non-KO_Rep2	41,996,644	40,378,554 (96.15%)	39,233,377 (93.42%)	51.42%	94.22%
non-KO_Rep3	40,846,342	39,084,181 (95.69%)	37,212,170 (91.10%)	51.89%	93.98%
lax1-KO_Rep1	41,233,466	39,345,996 (95.42%)	38,248,489 (92.76%)	52.11%	93.21%
lax1-KO_Rep 2	43,755,668	41,944,874 (95.86%)	40,806,727 (93.26%)	51.75%	94.09%
lax1-KO_Rep 3	40,663,756	39,025,113 (95.97%)	38,014,647 (93.49%)	52.14%	94.10%
